# Supplementary figures and images for: Computational and experimental analysis of the glycophosphatidylinositol-anchored proteome of the human parasitic nematode Brugia malayi
Source: PLoS One. 2019 Sep 12;14(9):e0216849. doi: 10.1371/journal.pone.0216849 (PMC6742230; doi:10.1371/journal.pone.0216849)

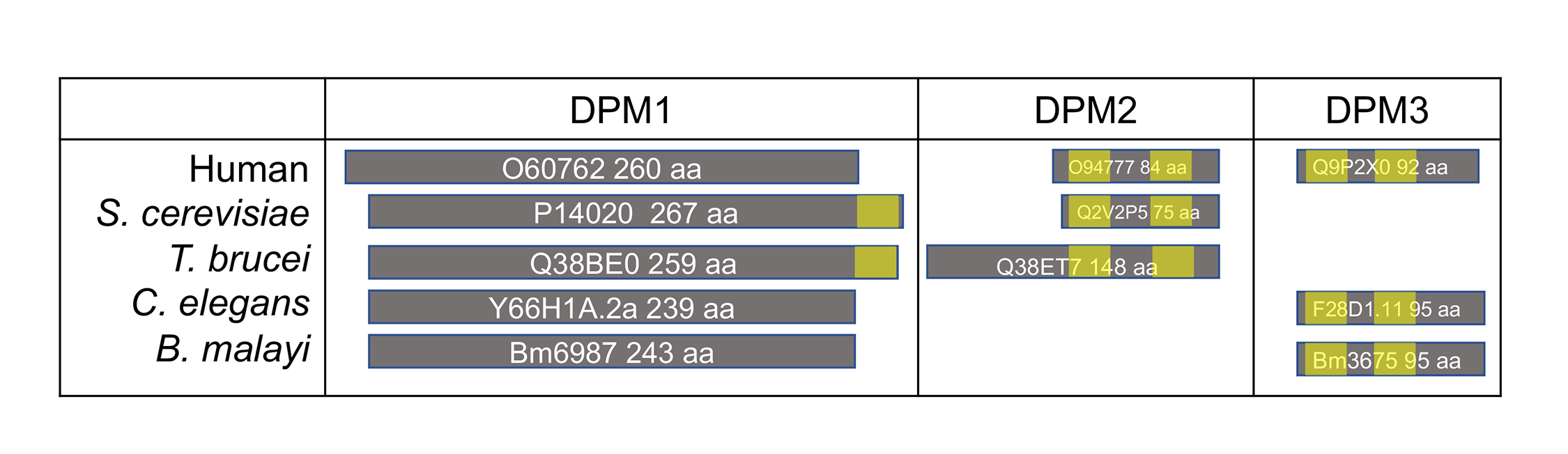

Supplement: S1 Fig — The DPM proteins for human, S. cerevisiae, T. brucei, C. elegans and B. malayi are shown with predicted transmembrane regions highlighted in yellow. Protein ID and protein length (aa = amino acids) are indicated for each. (TIF) [file pone.0216849.s001.tif]

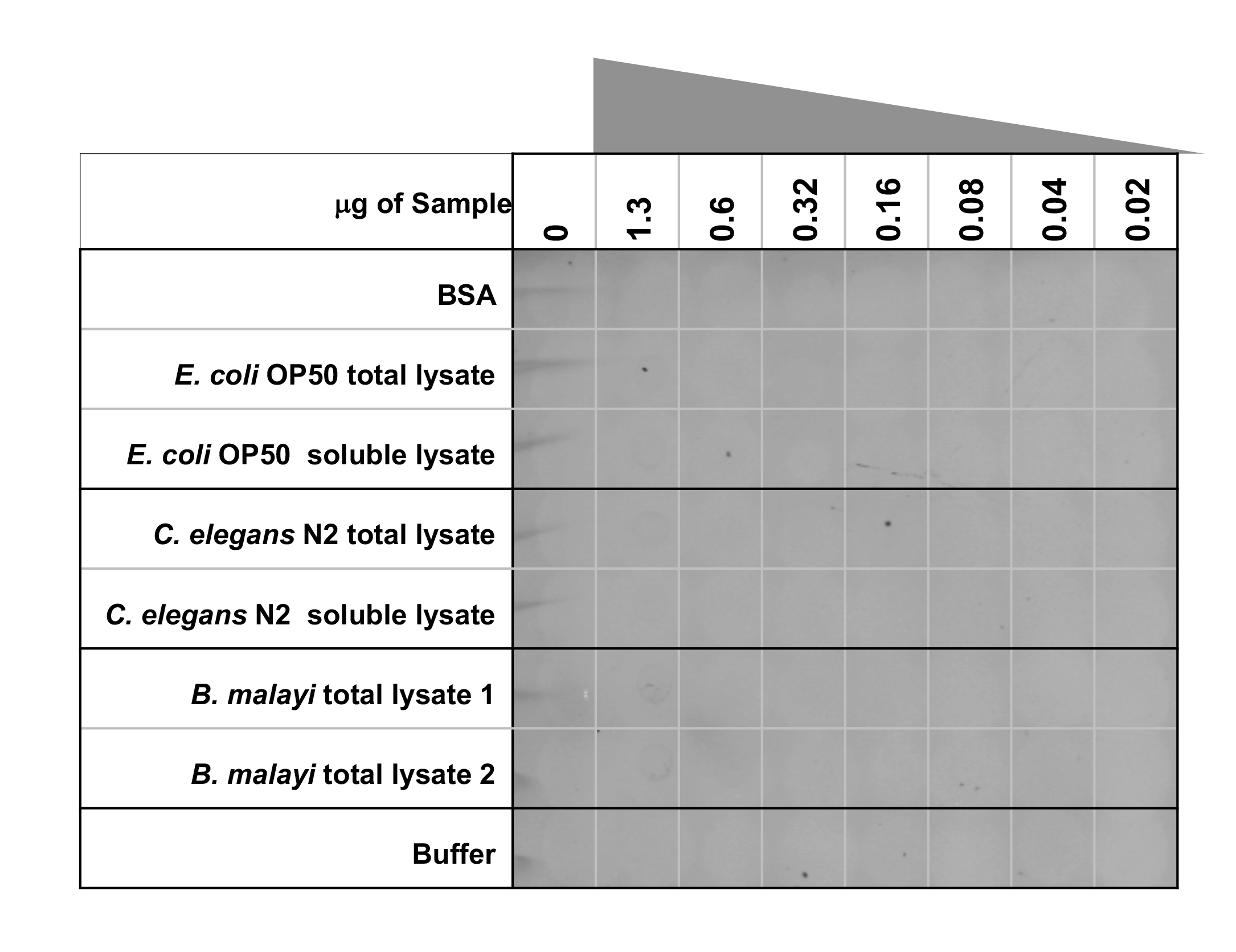

Supplement: S2 Fig — B. malayi and C. elegans lysates show no visible background signal or autofluorescence when FLAER reagent is not present. Samples: BSA, E.coli OP50 total and soluble lysate, C. elegans N2 total and soluble lysate, B. malayi total lysate 1: B. malayi total lysate mock control, B. malayi total lysate 2: B. malayi surface PI-PLC treated total lysate. After 1:1 series dilution, samples were spotted on to nitrocellulose membrane and incubated overnight in buffer. (TIF) [file pone.0216849.s002.tif]

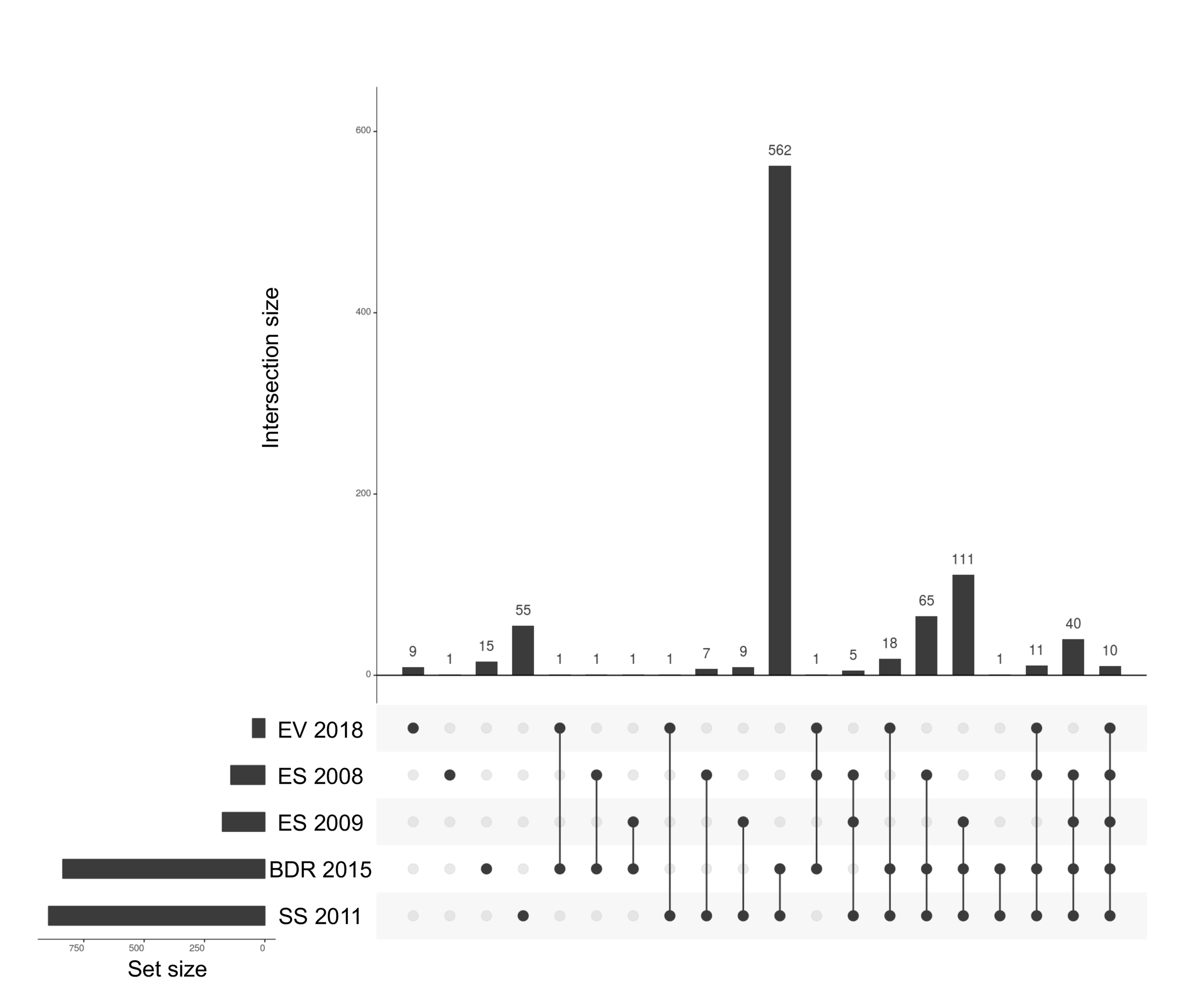

Supplement: S3 Fig — EV 2018 [54] is an extracellular vesicle proteome. ES 2008 [55] and ES 2009 [56] are excretory and secretory proteomes and BDR 2015 [57] is body wall, digestive tract and reproductive tract proteomes. SS 2011 [58] is a stage specific proteome set. Proteins that are exclusive to a set are designated with a single dot. Proteins that are found in multiple sets have dots that are linked with lines. The set size shown on the left is the number of proteins identified in the different proteome datasets that match the 1012 proteins identified in this study. (TIF) [file pone.0216849.s003.tif]
